# Axenfeld–Rieger syndrome: orthopedic and orthodontic management in a pediatric patient: a case report

**DOI:** 10.1186/s13005-022-00329-y

**Published:** 2022-07-08

**Authors:** Angela Pia Cazzolla, Nunzio Francesco Testa, Francesca Spirito, Michele Di Cosola, Alessandra Campobasso, Vito Crincoli, Andrea Ballini, Stefania Cantore, Domenico Ciavarella, Lorenzo Lo Muzio, Mario Dioguardi

**Affiliations:** 1 Department of Clinical and Experimental Medicine, Università degli Studi di Foggia, Via Luigi Rovelli, 50, 71100 Foggia, Italy; 2 Department of Basic Medical Sciences, Neurosciences and Sensory Organs, “Aldo Moro” University of Bari, Piazza Giulio Cesare 11, 70124 Bari, Italy; 3 Department of Precision Medicine, University of Campania “Luigi Vanvitelli”, 80138 Naples, Italy; 4 Faculty of Dentistry (Fakulteti i Mjekësisë Dentare-FMD), University of Medicine, 1001 Tirana, Albania

**Keywords:** Case report, Axenfeld–Rieger syndrome, Rieger anomaly, Orthodontic treatment

## Abstract

Axenfeld–Rieger Syndrome (ARS) is a rare autosomal dominant genetic disease with considerable expressive variability, characterized by ocular and non-ocular manifestations, cardiovascular, mild craniofacial abnormalities and dental malformations. Current data report an incidence of Xenfeld-Rieger syndrome in the population of 1: 200,000.

The case described is that of a 14-year-old female patient whose ARS is suspected and investigated following a dental specialist visit for orthodontic reasons, acquired the patient’s family and clinical data following a medical approach multidisciplinary, we proceed to the orthodontic involved the use of the Rapid Palatal Expander (RPE) and a fixed orthodontic treatment.

The aim of this study is to report the case of the orthopaedic and orthodontic treatment in a patient affected by ARS and with facial dysmorphism and teeth anomalies associated to ocular anomalies.

## Introduction

Axenfeld–Rieger Syndrome (ARS) is a rare autosomal dominant genetic disease with considerable expressive variability [1], characterized by ocular and non-ocular manifestations (cardiovascular, mild craniofacial abnormalities and dental malformations). In some cases, patients may have short stature, mental retardation and finger malformations [2–6]. An abnormal migration and differentiation of neural crest cells are considered responsible for anomalies in ocular, craniofacial and dental development. In this syndrome, some authors trace various clinical conditions such as Axenfeld’s anomaly, Rieger’s anomaly and Rieger’s syndrome, even if analysis of the genetic profiles suggests different pathological conditions [7].

It is a heterogeneous disorder inherited in an autosomal dominant manner and shows significant expressive variability both between families and within the same family [8] .

Current data report an incidence of Xenfeld-Rieger syndrome in the population of 1: 200,000 while there is no definitive data on a gender and racial prevalence [9, 10]. The mutation in genes of two transcription factors, FOXC1, FOXC2, FKHL7, PANCR and PITX2 [11–14], represent almost half of the known cases while the genetic defect is not known in 60%.

Defects of the ectodermal tissue with involvement of the cells of the neuronal crest, which arise at the 3rd trimest of pregnancy, can be the cause of the facial morphological abnormalities of the maxilla, jaw, hypertelorism, teeth, saddle and non-involution of the skin periumbilical [15].

Clinical aspects may concern ocular alterations (malformations of the ocular anterior segment) and pathologies such as: heterochromia, aniridia, coloboma of the iris [16], an a prominent anteriorly displaced Schwalbe’s line. Pupil abnormalities in form, position and number occur. Additional ocular problems of ARS cause increased intraocular pressure resulting in glaucoma in approximately 50% of children with ARS. Other ocular anomalies include corneal opacities, lens defects, conjunctival xerosis, blue sclera, chroidal hypoplasia and retinal detachment [17]. Craniofacial malformations include maxillary hypoplasia hyperplastic maxillary, cleft palate, mandibular protrusion with anterior open bite, hypertelorism a broad nasal bridge and an enlarged sella turcica. The face appears to be flattened, with a prominent forehead and a flat, broad nasal root. Widely spaced eyes, a broad flat bridge of the nose and / or a protruding lower lip. Dental alterations include hypodontia or partial anodontia, microdontia, taurodontia, abnormally shaped teeth, peg-shaped teeth, enamel hypoplasia, hyperplastic maxillary labial frenum [18].

In addition, the presence of periumbilical skin, ear malformations with conductive deafness, anomalies of the pituitary gland, congenital heart defects renal malformation, kyphosis, scoliosis, maldevelopment of the sternum, alterations of the extremities, myotonic dystrophy with muscular atrophy, growth retardation, lypodystrophy, lack of vision, drooping eyelids may be present.

Some patients present mental retardation with problems with learning, language and behavior.

There are few reports concerning case presentations of patients with ARS and few papers [19–22] concerning orthopedic-orthodontic treatment relative to the patients with this syndrome. The aim of this study is to report the case of the orthopedic and orthodontic treatment in a patient affected by ARS and with facial dysmorphism and teeth anomalies associated to ocular anomalies.

## Case report

An Italian female patient of 14 years came to our observation from the Ophthalmology Department of the University of Bari for dental evaluation and orthodontic therapy to correct problems of bad occlusion with misalignment of the teeth.

The patient was examined clinically and thoroughly and a family and medical history recorded. Clinical and medical findings led to the clinical diagnosis of Axenfeld-Rieger syndrome.

**Fig. 1:**
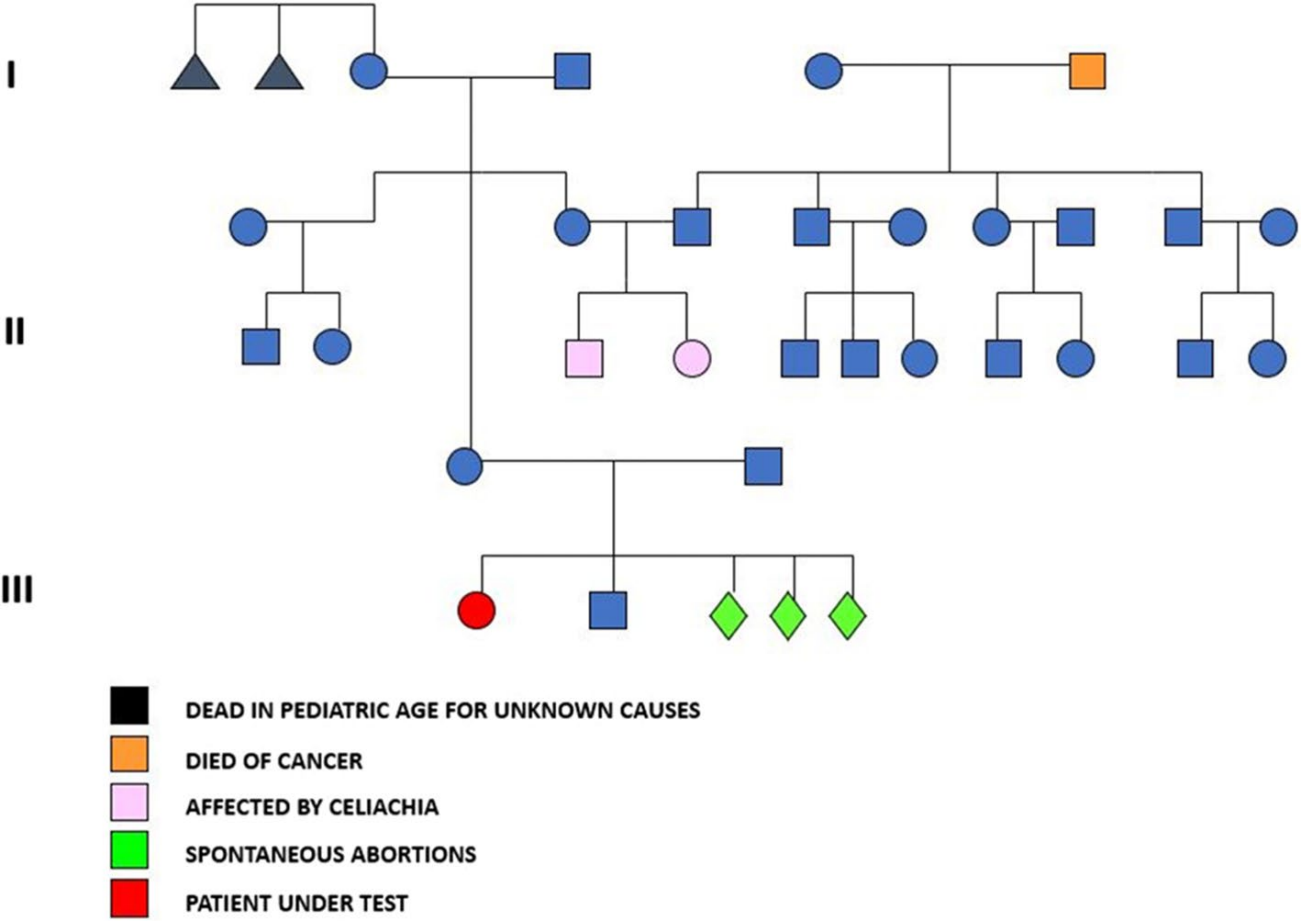
Pedigree of three generations of the family of the patient with Axenfeld-Rieger Syndrome

The family pedigree is shown in Fig. 1.

Family tree show that most of the family does not affected by Reiger syndrome. The cousins ​​are affected by celiac disease, moreover the family history shows how the parents had 3 spontaneous abortions.

**Fig. 2:**
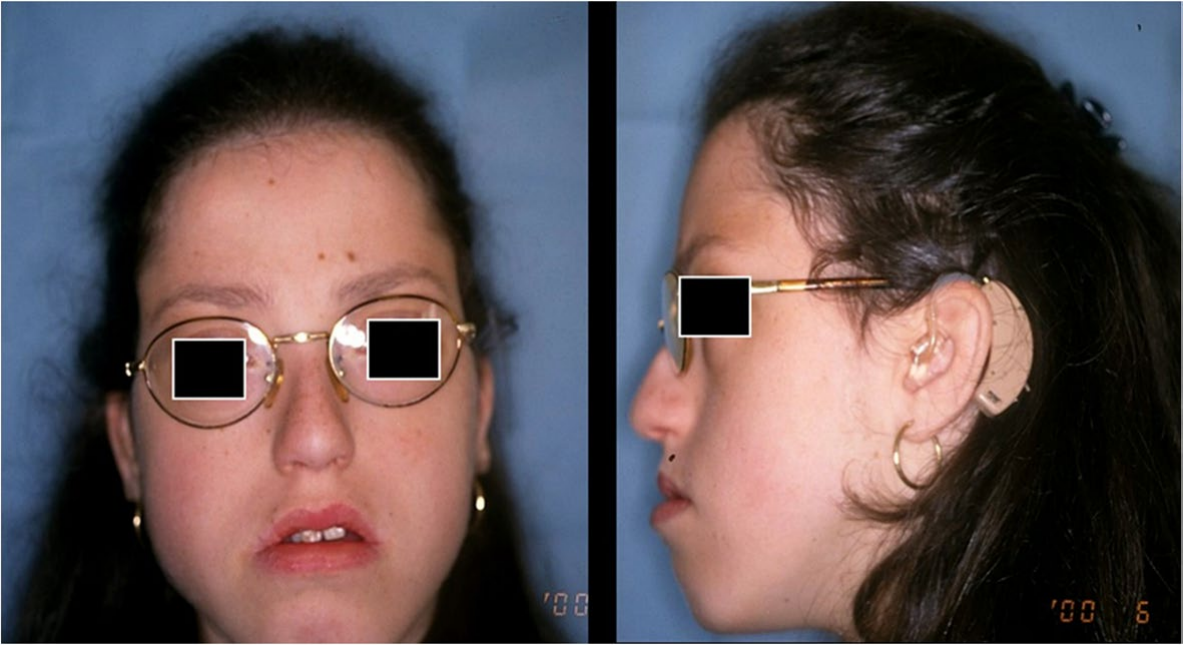
Extraoral photo (**a**) frontal view before treatment; **b** lateral right view before treatment

**Fig. 3:**
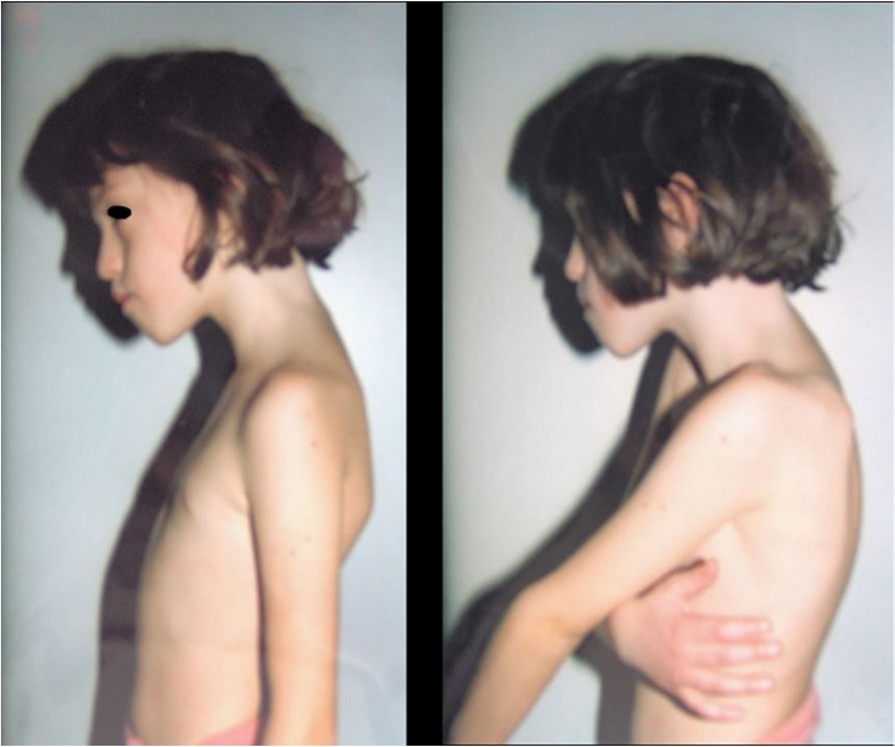
Profile image of the patient at 8 years of age with evidence of joint laxity and lordosis

Specialist medical examinations report the following clinical data: psycho-intellectual and motor retardation, skeletal age retardation with vertebral lordosis and joint laxity (Figs. 2 and 3), speech delay, severe bilateral sensorineural hearing loss and the presence of bilateral congenital glaucoma.

**Fig. 4:**
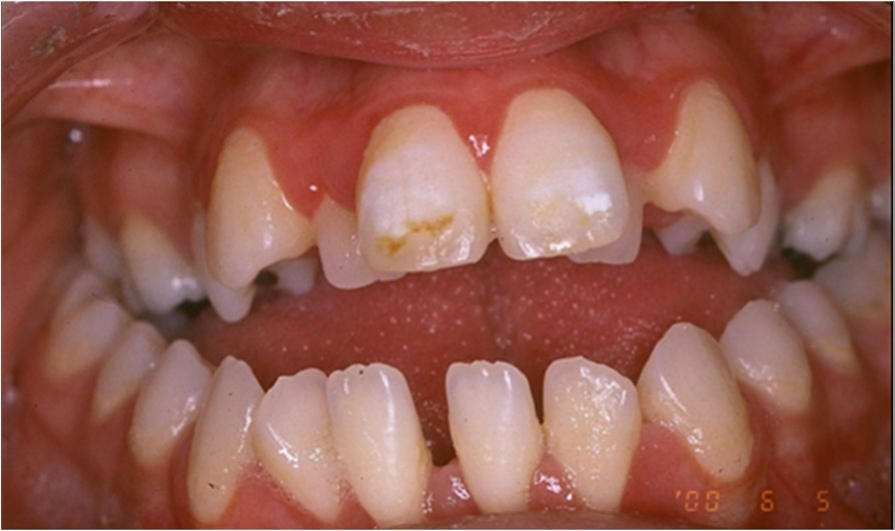
Presence of open bite with enamel hypoplasia on elements 1.1 and 2.1

**Fig. 5:**
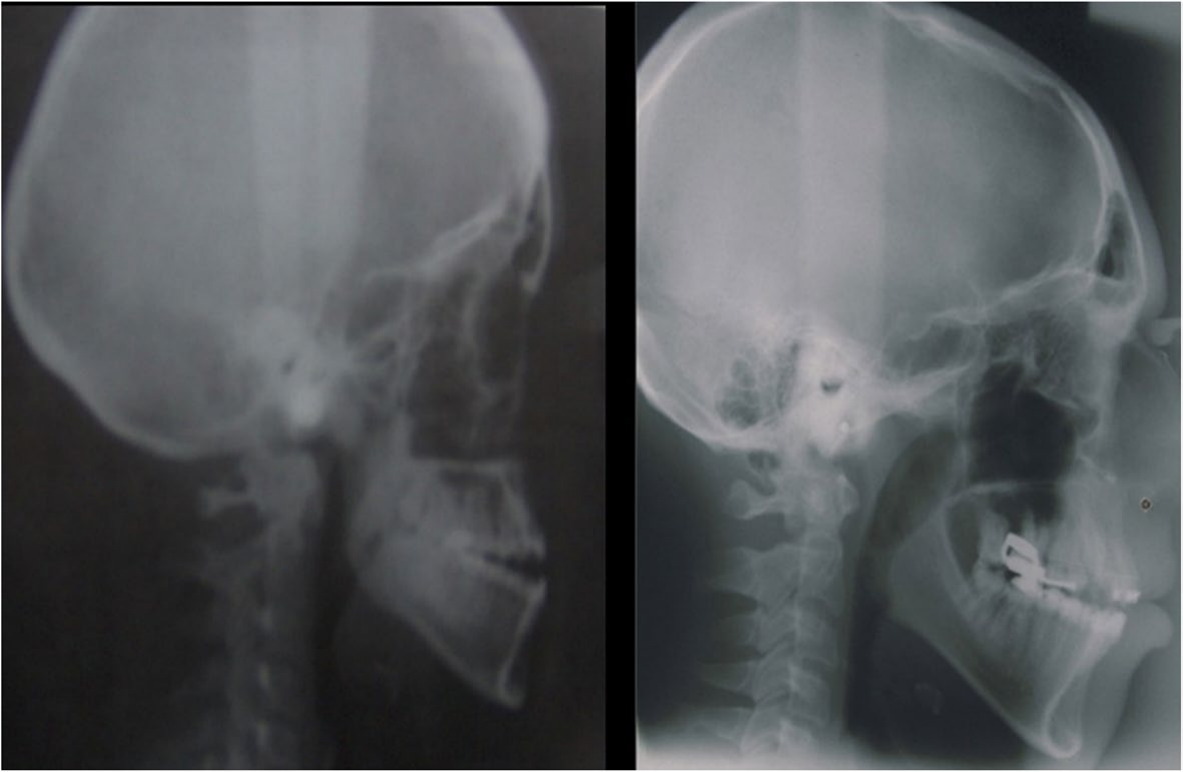
**a-b** Latero-lateral teleradiography of the head before treatment; Latero-lateral teleradiography of the head with RPE

The intra-oral examination shows the presence of open bite with enamel hypoplasia affecting the upper central incisors, lower diastema due to lingual thrust with non-coincident intericisive lines (Fig. 4) the presence of conoid-like teeth and tarurodontism with some elements that radiographically showed signs of ankylosis, transverse contraction of the upper jaw and dental crowding. The laterolateral teleradiography (L-L rx) (Fig. 5a) and the relative cephalometric analysis according to Jarabak revealed the presence of a skeletal Class III (subspinale(A)-nasion(N)-suprmanetlae (B) angle ANB = − 5°), skeletal open-bite, hyperdivergence, mandibular growth with vertical growth pattern (Table 1). The aim of the treatment was to correct or at least to avoid the worsening of the skeletal relationship between the upper and lower jaws and to resolve the crowding in the upper arch. The therapy for malocclusion involved the use of the Rapid Palatal Expander (RPE) and a fixed orthodontic treatment.

**Table 1:** Cephalometric analysis according to Jarabak

Cephalometric L-L study	Normal value	Before treatment	After treatment
Sagittal skeletal relationships (°)			
SNA	82 ± 2	84	84
SNB	80 ± 2	89	90
ANB	2 ± 2	−5	−6
Vertical skeletal relationships (°)			
Ar-S-N	122 ± 5	120	120
S-Ar-Go	143 ± 6	150	152
Ar-Go-Me	120 ± 5	135	135
Ar-Go-N	50 ± 2	47	45
N-Go-Me	70 ± 3	88	88
S + Ar + Go	396 ± 2	405	407
S-N/Go-Me	32 ± 5	41	40
Vertical skeletal relationships (mm)			
N-Me		116	118
S-Go		65	66
% Jarabak	59–63%	56%	55%
Sagittal plane (mm)			
S-N	71 ± 3	50	53
Go-Me	71 ± 5	57	58
Cranial base length (mm)			
S-N	71 ± 3	50	53
S-Ar	32 ± 2	22	24
Mandibolar component (mm)			
Ar-Go	44 ± 5	33	35
Go-Me	71 ± 5	57	58
Dentoalveolar component (°)			
U1toSN	102 ± 2	112	97
L1toGoMe	90 ± 3	84	85
Interincisal angle	130 ± 5	138	125

After a plurispecialized medical examination, an oral hygiene and prophylaxis program with recurrent medical checks, the patient underwent conservative therapies.

Then the patient was subjected an orthopedic-orthodontic treatment divided in two steps:

- Orthopedic treatment with RPE with a protocol of two activations per day for 15 days in order to correct the transversal contraction of the upper jaw, maintained for 1 year.

Therefore, the fixed orthodontic treatment was started.

**Fig. 6:**
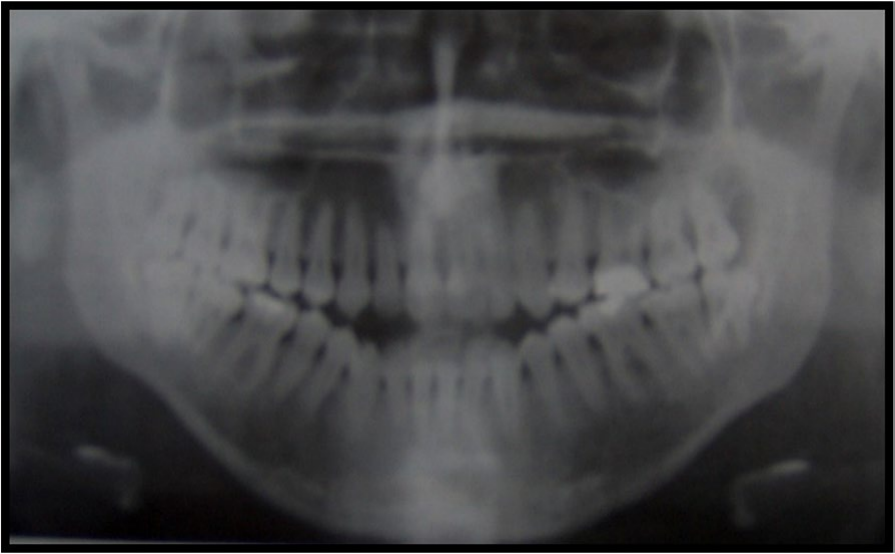
Panoramic X-ray

- Orthodontic treatment of the upper arch with Straight Wire technique (orthodontic archwire sequence: NiTi .014; NiTi .016; NiTi .018; NiTi .016x.022; Stainless steel .016x.022) was performed. This treatment allowed the dental alignment, increased the space in the dental arch to solve dental crowding and improved the cross-bite and the open bite through strategic positioning of the brackets (Figs. 5b and 6).

## Discussion

The malformations of the anterior chamber of the eye and the presence of dental anomalies are fundamental elements that characterize ARS. The only involvement of the eyes is called Rieger anomaly or Axenfeld anomaly or hypoplasia of the iris, while the presence of symptoms and of extraocular signs preponderate towards the ARS syndrome. These 2 conditions can be superimposed suggesting a single pathological entity, but genetic analyzes on patients diagnosed with Rieger and ARS anomaly provide information on the presence of 2 slightly different conditions.

The disease is autosomal dominant with variable penetrance; in our case report the genealogical survey did not show the presence of relatives diagnosed with ARS even if they showed a predisposition to neoformative pathologies [23], therefore a reduced penetrance of the syndrome is assumed. Another feature is the absence of dental agenesis while only dental anomalies (enamel hypoplasia localized on some teeth) were present. The shape of the sella turcica in the present case was apparently normal, an open bite and a skeletal class III was present (Fig. 5). In fact, maxillary hypoplasia was observed in about 90.5% of cases, with a concave facial profile and a flat medial face [24]. This patient did not have the classic dental presentation of ARS, such as the lack of central and lateral incisors and canines described by other authors. In fact, the treatment only involved the use of RPE and fixed orthodontic treatment for alignment of the dental elements. However, the fundamental orthodontic treatment to partially respond to aesthetic needs can be problematic due to the presence of dental anomalies (short and curved roots, ankylosis and poor vertical dimension of the bone alveolar ridge). Some authors recommend a post adolescent orthodontic surgical approach [22]. Patients with rare diseases often presented specific orthodontic risks associated with the systemic disorders. Therefore, orthodontists must understand the orthodontic risks associated with these conditions and must carefully design an orthodontic treatment plan to minimize the risks. In patients with ARS, the risks of orthodontic treatment have not been clear in part because few cases of orthodontic treatment have been reported [21]. A priority aspect for this type of patient is the maintenance of correct oral hygiene as for all patients suffering from rare diseases [25–31] in order to safeguard the periodontal condition having a reduced size of the mandibular and maxillary bone crest and the presence of short roots with little presence of adherent gingiva [32, 33]. These factors can determine the premature loss of tooth following periodontal disease, furthermore the maintenance of periodontal health is fundamental for implant rehabilitation in sectors with agenesis or where there has been the loss of dental elements, not easy to plan as described by Pirih et al. for the reduction of bone thickness [34].

Waldron et al. report the presence of osteopenia of the bones in association with this syndrome (reduced calcification, decreased density or reduced bone mass) with predisposition to osteoporosis, recommending the intake of vitamin D and physical exercise for the prevention of this complication [35].

Dental and orthodontic treatment plays a fundamental role so that patients suffering from the syndrome improve the functional and phonetic aspects compromised by malocclusion and hypodontics. Furthermore, improving the aesthetics of the smile as well as the support of the upper lip and the general appearance helps patients in their relationship life. In fact, delaying aesthetic dental rehabilitation can lead to discomfort in young patients with impaired social life at school age [19].

### Genetic aspects

The genes, mainly involved in the mutation responsible for 50% of ARS cases, are forkhead box C1 (FOXC1) and pituitary homeobox 2 (PITX2).

DNA mutations involving PITX2, located at 4q25 [36], result in ARS in which patients have ocular phenotypes often seen with craniofacial and dental abnormalities, while mutations in FOXC1 located at 6p25 result in ARS defined by ocular phenotypes observed with cardiovascular defects and sensorineural hearing loss.

The presence of the mutation of these two genes was investigated in a multicentric study conducted by Souzeau et al. which included 34 individuals from 18 families: FOXC1 variants were present in 64.7% of individuals and PITX2 variants in 35.3% of individuals [37].

Other genes have however been taken into consideration and investigated in the pathogenesis of ARS; in fact, there seems to be an involvement of a sequence between the PITX2 gene and the noncoding PANCR gene as reported by Qui et al. [13] whose mutation is involved in congenital glaucoma and other forms of glaucoma [38]. heterozygous missense variant in the PRDM5 gene localizes to human chromosome 4q26 is reported by Micheal et al. associated with PITX2 [39].

While more and more frequently mutations of the FKHL7 gene located at 6p25 is reported in the association ARS [40–43], also some sporadic cases of ARS have been related to deletion of the PAX6 gene (still controversial and to be definitively demonstrated [44]) at 11p13 and deletion of the 16q23-q24 region; a second locus for Rieger syndrome located on chromosome 13q14 has also recently been identified [45].

## Conclusions

Early diagnosis of ARS is essential to avoid complications caused by glaucoma, and patients should also undergo repeated eye, pediatric, physiotherapy and speech therapy checks to maintain psychophysical health and for a more favorable insertion into social life. While, the dental management of these patients must be aimed at the maintenance of dental hygiene and must be aimed to the morpho-functional restoration with a multidisciplinary approach that includes orthodontic, oral and maxillofacial surgery, implant surgery and restorative treatments together with speech and language therapy.

## Data Availability

Not aplicable.
